# Choosing the Correct Internal Reference Redox Species for Overcoming Reference Electrode Drift in Voltammetric pH Measurements

**DOI:** 10.1021/acselectrochem.5c00138

**Published:** 2025-06-13

**Authors:** Nafiz B. Biswas, Tania Read, Katherine J. Levey, Julie V. Macpherson

**Affiliations:** † Department of Chemistry, 2707 University of Warwick, Coventry CV4 7AL, United Kingdom; ‡ Leiden Institute of Chemistry, Leiden University, Einsteinweg 55, 2333 CC Leiden, The Netherlands

**Keywords:** Reference electrode drift, internal reference, voltammetric pH electrode, proton coupled electron transfer, square wave voltammetry, Stow-Severinghaus carbon dioxide electrode, boron doped diamond electrode, apparent peak shifts

## Abstract

Reference electrode (RE) drift is a common problem when electrodes are used for pH determination, especially over extended periods of time or in complex media. For voltammetric pH measurements, one method to mitigate against RE drift is to add a second pH insensitive redox species (the internal reference, IREF) and measure the difference in peak potential, *E*_diff_, between the signal associated with the pH sensitive species, *E*_pH_, and IREF, *E*_IREF_. This work strategically explores how to choose the correct IREF species. For these studies, a quinone-functionalized boron doped diamond (BDD-Q) electrode is employed as the pH sensing electrode over the pH range of 4–9. To avoid errors in reporting of the real *E*_pH_ and *E*_IREF_ values, there must be a minimum separation between the two peaks. Moreover, the distance on the potential axis between a peak and that of the current response pertaining to water electrolysis must also be considered. For the BDD-Q pH electrode, an operable potential window for IREF is established and the IREF redox species, hexachloroiridate (IrCl_6_^2–/3–^), is determined to be most appropriate, showing an ∼0.08 pH error over the pH range of 4–9 and reducing to ∼0.02 pH error over the pH range of 6–8. The use of *E*_diff_ is further assessed via the voltammetric measurement of dissolved carbon dioxide (CO_2_) in a Stow-Severinghaus arrangement over the partial pressure range of 30.4–152.0 mmHg. The *R* ^2^ linearity of the calibration line (=0.998) is shown to be equivalent and in agreement with theory when plotting either *E*_diff_ or *E*_pH_ versus CO_2_ partial pressure. This data bodes well for the use of *E*_diff_ as a measurement signal in Stow-Severinghaus dissolved CO_2_ transcutaneous sensors, where continuous measurement of pH over several days is required.

## Graphical abstract

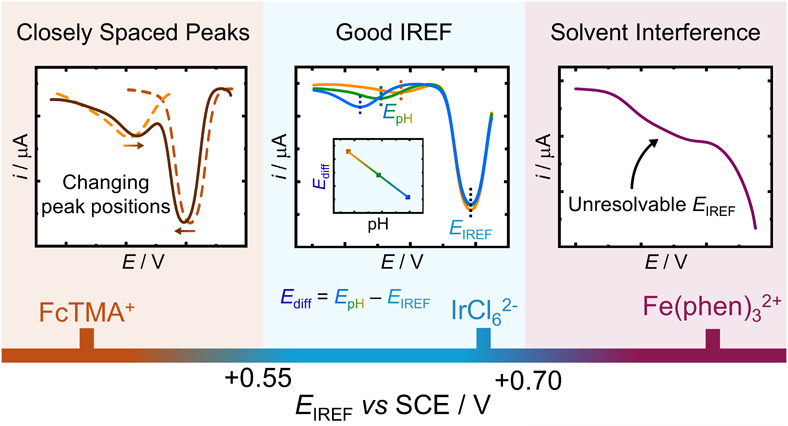

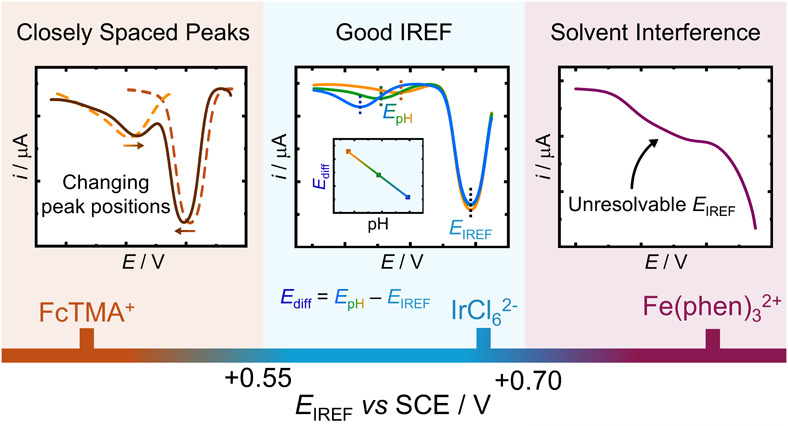

## Introduction

In electroanalysis, attention is often focused on the sensing (working) electrode, but this means the vital role of the reference electrode (RE) can be overlooked. The RE provides a stable, well-defined potential against which the potential at the sensor electrode is controlled and measured.^1^ RE drift is a significant challenge in any electrochemical sensor system, where an output voltage is the measurement signal. This includes both the more common potentiometric sensor and the voltammetric sensor, with the latter operating under conditions in which the potential for a characteristic (often peak) current is the measurement signal of interest.

RE drift can arise for many reasons. In systems which use glass fritted REs such as the silver, silver-chloride electrode (Ag|AgCl|Cl^–^_(aq)_) and the saturated calomel electrode (SCE),^2^ mechanical damage to the frit can result in loss of potential determining chloride ions from the filling to the test solution.^3^ Extended use, especially in real water samples, can also result in clogging of the frit due to precipitate or biofilm formation.^4^ For sensing devices where miniaturization and ease of construction is important, a Ag|AgCl quasi reference electrode (QRE) is typically used. This consists of a Ag wire coated in AgCl, which sits directly in the solution of interest. Whilst the rapid rate of dissolution of the sparingly soluble AgCl controls the concentration of Cl^-^ in the vicinity of the AgCl (especially in Cl^-^ free solutions),^5^ this reference electrode is more susceptible to local changes in the analyte solution.^6^

One of the most important solution parameters measured electrochemically for both laboratory-based and real-world solutions is the pH. pH sensors typically output a characteristic potentiometric voltage using, e.g., glass pH sensitive electrodes^7^ or metal oxides^8^ or voltammetrically using, e.g., quinone-based systems that undergo proton coupled electron transfer (PCET).^9^ pH can also be used to inform indirectly on dissolved carbon dioxide (CO_2_) concentrations via measurement of the pH change in a bicarbonate buffer solution, the Stow-Severinghaus electrode.^10–12^ Dissolved CO_2_ measurements also have a wide range of applications ranging from environmental (fresh, sea, and aquarium water monitoring) to corrosion control, beer brewing, and medical care.^13^ Due to RE drift, the frequency of calibration required in such settings often precludes their use for long-term applications such as, e.g., continuous blood-gas monitoring over the period of a week.^14^

For potentiometric pH measurements, as only a voltage is measured, the instrumentation is simpler; however, this means there is far less scope for adapting the actual measurement protocol to build in methods to account for RE drift. In contrast, for voltammetric pH measurements, it has been demonstrated as early as the 1980’s^15^ how addition of a pH independent redox species, referred to as the internal reference (IREF), can be used as a possible RE drift mitigation strategy.^15^ Addition of an internal standard (or reference redox species) is common for non-aqueous systems, but the reasoning is different and is due to the difficulties of finding a suitable reference electrode in such solvent systems.^16^

For the aqueous system, as the pH changes, the potential associated with the peak current of the pH dependent species moves with respect to that of IREF, which, ideally, remains fixed in position. The difference in potential between the two signals, referred to as *E*_diff_, is used as a metric for determining solution pH.^15,17^ If RE drift occurs, while the peak currents for both the pH sensitive and pH insensitive species shift, *E*_diff_ remains unaffected and an accurate measurement of pH is obtained. This concept is schematically shown in Figure 1. Square wave voltammograms (SWVs) are typically used in analysis due to the sharper peaks obtained in the current–voltage response compared to those in cyclic voltammograms (at macroelectrodes).^18^

**Figure 1:**
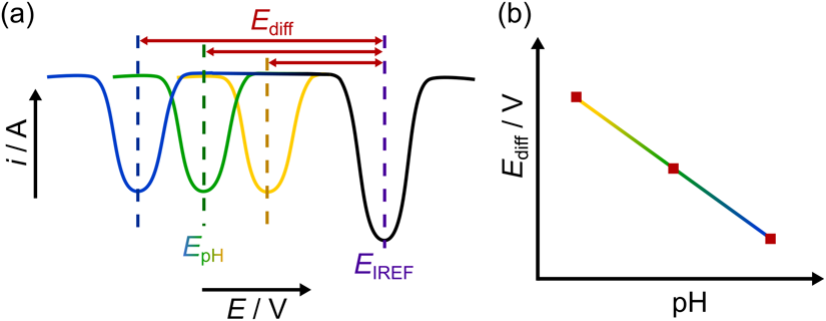
(a) Schematic depicting SWV scans in solutions of decreasing pH (from left to right); *E*_pH_ is the peak potential of the pH dependent peak whilst *E*_IREF_ corresponds to the peak potential for the pH independent, IREF species. (b) *E*_diff_ plotted vs pH to form a calibration line.

In the original work,^15^ either a pH sensitive conducting polymer (polyaniline) or quinone containing species (chloranil) was immobilized on Pt wires in the presence of Nafion encapsulated Ru­(bipy)_3_^2+^ (IREF). For polyaniline, pH values could only be measured up to ∼8 and reproducibility issues were raised. Whilst the chloranil system was found to be more stable, it was necessary to consider the oxidation/reduction state of the Pt and pH values could only be measured up to pH ∼6.^15^ Further studies extended this concept to self-assembled monolayers or drop cast “inks”, containing both the pH sensitive and pH insensitive molecules.^17^ This work, and others that followed, used ferrocene (Fc) or Fc derivatives as IREF, immobilized on macro-^19^ and micro-sized^17^ gold electrodes, glassy carbon electrodes,^20^ and screen printed graphite electrodes.^21,22^ However, each of these studies presented its own complications. In the original gold microelectrode work, the Fc IREF potential moved by ∼50 mV (estimated from Figure 4 in ref 17) over the pH range of 1–8, which is almost a pH unit given that the Nernstian pH responses shift by 59 mV/pH at 298 K.^23^ By decreasing to a pH range of 5–9, the variation in IREF was reduced to 15 mV (estimated from Figure 3 in ref 19), and by moving to an even narrower range (pH 7.30–7.50), the IREF variation was reduced further to 7 mV.^22^ This work was expanded by using two working electrodes, one as the sensor and the other to correct RE drift (the calibration electrode). However, the peak potential of the calibration sensor varied by 50 mV over the pH range of 2–13 (estimated from Figure 2 in ref 24). In other work, the Fc and quinone groups were combined into one molecule and drop cast onto glassy carbon; however, a reduced linearity in the pH–*E*_diff_ calibration slope (*R* ^2^ = 0.969) was found.^20^ Finally, in only one other study, have different redox couples been explored. Here, two redox polymers containing an osmium redox couple (IREF) and a pH sensitive phenothiazine moiety were electrochemically deposited on a gold microelectrode; no quantification was made of *E*_IREF_ stability.^25^

Given the prior literature, which shows non-negligible variations in the IREF potential with pH, in this paper, we explore the different factors that must be considered when identifying the ideal IREF redox species for voltammetric pH measurements. For these studies, we use boron doped diamond (BDD) electrodes functionalized in defined locations with a very robust form of oxygen-terminated sp^2^ carbon,^9,26^ given they have shown considerable promise as voltammetric pH sensors. The sp^2^ carbon regions contain surface integrated quinones (Q’s), which undergo 2e^–^2H^+^ PCET up to a pH value associated with the first pK_a_ of the quinone (typically ca. pH = 12).^9,27^ The electrodes show Nernstian behavior^9^, in both buffered and unbuffered solutions.^30^ Whilst we apply the study to quinone-functionalized boron doped diamond (BDD-Q) pH electrodes, operating over the pH range of 4–9, the principles established will apply to any voltammetric pH sensor, over the required pH range of interest. This pH range covers environmental and medical fluids including seawater,^31^ fresh water,^32^ and blood.^33^ The IREF *E*_diff_ method is further demonstrated by using, for the first time, the BDD-Q pH electrode as a Stow-Severinghaus dissolved CO_2_ sensor. The use of the IREF methodology for measuring pH and dissolved CO_2_ is compared against the conventional reference electrode approach to determine its viability for use with pH/dissolved CO_2_ BDD-Q sensors.

## Experimental Section

### Solutions and Chemicals

All solutions were prepared using ultra-pure Milli-Q water (Millipore Corp.) with a resistivity of 18.2 MΩ cm at 25 °C. Oxidative acid cleaning of the BDD used potassium nitrate (KNO_3_, 99%, Fisher Scientific) and concentrated sulfuric acid (H_2_SO_4_, 98%; Sigma-Aldrich).^26^ Reagecon buffers (Reagecon Diagnostics Ltd., Calibre Scientific) of pH 4.00, 6.00, 7.00, 8.00, and 9.00 (±0.01) were used to perform the BDD-Q pH tests. Carmody buffers were made in the pH range of 4–9 for electrode calibrations and were prepared using boric acid (H_3_BO_3_, 99.97%; Sigma-Aldrich), citric acid (C_6_H_8_O_7_, ≥99.5%; Sigma-Aldrich), and tertiary sodium phosphate (Na_3_PO_4_, ≥95%; Sigma-Aldrich).^34^ For dissolved CO_2_ measurements, 20 mM potassium bicarbonate (KHCO_3_, ≥99.95%, Fisher Scientific; Sigma-Aldrich) was used. The IREF redox species investigated were ferrocenylmethyltrimethylammonium, FcTMA^+^, hexafluorophosphate (made in house),^35^ potassium hexachloroiridate­(IV), IrCl_6_^2–^ (99.99%, Merck; Sigma-Aldrich), and tris­(1-10-phenanthroline) iron­(II), Fe­(phen)_3_^2+^, sulfate (0.25 M, Fisher Scientific). Potassium chloride (KCl, ≥99%, Fisher Scientific) with concentrations up to 0.2 M and KNO_3_ with concentrations up to 0.09 M were used as supporting electrolyte for electrochemical measurements. Solution pH was measured using a glass pH probe (InLab Expert Pro ISM, SevenEasy, Mettler Toledo). The probe was calibrated using a four-point calibration, employing NIST standard pH solutions (pH 2.00 ± 0.01, pH 4.01 ± 0.01, pH 7.00 ± 0.01, and pH 10.00 ± 0.01; Sigma-Aldrich), as per manufacturer guidelines.

### Electrode Manufacture

The BDD was cut from a 357 μm thick, free-standing wafer of polycrystalline, electroanalytical grade BDD (electrode E in ref 36), Element Six Ltd, Oxford. The growth face was polished to <5 nm RMS surface roughness and used as the electrode surface. 1 mm diameter BDD cylinders were cut from the wafer using a 355 nm Nd:YAG 34 ns laser micromachining system (E-355H-ATHI-O system, Oxford Lasers). Oxidative acid cleaning, using boiling concentrated H_2_SO_4_ saturated with KNO_3_, was used to remove any loosely bound sp^2^ carbon introduced after the micromachining process.^26^ The cylinders were annealed electrode face-down in air at 600 °C for 5 h to significantly reduce sp^2^ carbon on the cylinder walls, arising from the laser cutting processing.^26^ Spatially controlled regions of sp^2^ carbon were laser micromachined into the electrode face of the cylinder, as described in detail in ref 9, before undergoing a second oxidative acid clean. 10 nm of titanium and 400 nm of gold were sputtered (Moorfields MiniLab 060 platform sputter/evaporator) onto the back (nucleation side) of the cylinders, which were annealed at 400 °C for 5 h in air, again electrode-face down, to ensure an ohmic contact. Finally, the cylinders were sealed in heat-pulled glass capillaries (o.d. 2 mm; i.d. 1.16 mm; Harvard Apparatus Ltd., Kent, UK) and polished to reveal the glass-sealed electrode as described in ref 37.

### Electrochemical Setup

All electrochemical measurements were conducted by using a CH1040a potentiostat (CH Instruments Inc., USA). All experiments were conducted at a temperature of 21 ± 2 °C. A BDD-Q electrode was used as the working electrode, either an SCE or silver chloride coated Ag foil (Ag|AgCl) as the RE, and a coil of platinum wire (diameter = 1 mm, ∼10 mm length immersed in solution) as the counter electrode. The Ag|AgCl RE was prepared by anodization of an annealed Ag foil (0.5 mm thick, Premion, Thermo Fisher Scientific, 99.9985%) cut to ca. 5 mm in width and 30 mm in length. Ag was held at +0.54 V vs SCE with a Pt mesh counter electrode (to provide a large surface area compared to the silver) in a saturated KCl solution for ca. 10 min or until the foil visibly changed color from silver to brown.^38^ Solutions containing the IREF redox species were made using concentrations of 100 μM, in up to 0.2 M KCl supporting electrolyte.

For SWV measurements, scans were carried out in the cathodic direction with an amplitude of 0.1 V, a step increment of 0.001 V, a frequency of 100 Hz, and a quiet time of 2 s. These settings had been previously determined as being suitable for pH measurements using the BDD-Q electrode.^30^ For all BDD-Q voltammetric measurements, six SWVs were recorded consecutively. The first scan was discarded, and the current of the remaining five was averaged to provide a mean current across the potential range. The mean SWV was smoothed using a Savitzky–Golay (adjacent-averaging) filter with a polynomial order of three over 21 data points to provide sufficient smoothing without compromising the data. As a SWV scan with a 1 V potential range and a frequency of 100 Hz has 1000 data points, the smoothing window equates to ∼2% of the data points. Voltages corresponding to the peak SWV current were determined from the mean SWV. MATLAB R2021b was used to average SWV scans and to find the peak potentials. The MATLAB code used can be found in Supporting Information (SI)-1. For electrode voltage vs pH calibrations, Reagecon buffer standards, Carmody buffers, or bicarbonate solutions (depending on the final system being measured) with pH values in the range of 4–9 were used. Linear regression analysis of a plot of SWV peak potentials vs solution pH (with pH values measured using a calibrated Metler Toledo glass pH probe) was made using Origin 2021b (OriginLab).

### Mass Flow Controller Setup to Control Dissolved CO_2_ Concentration

Mass flow controllers, MKS Instruments, 100 standard cubic cm per minute (sccm), were used to control the partial pressure, pCO_2_, flowing into the 20 mM bicarbonate solution. CO_2_ and Ar were bubbled through the solution to produce pCO_2_ values in the range of 30.4–152.0 mmHg. Parafilm (Amcor) was used to seal the electrochemical cell and limit evaporation of solution. pCO_2_ values in the range of 35–48 mmHg are typical for blood.^39,40^ pCO_2_ values were calculated using eq 1:

$${\mathrm{pCO}}_{2}=760\;\mathrm{mmHg}\;\%{\mathrm{CO}}_{2}$$ 1

where 760 mmHg is atmospheric pressure and %CO_2_ is the sccm of CO_2_ compared to the total sccm gas flow through the cell (Ar and CO_2_).

## Results and Discussion

Figure 2a displays the SWV response of the BDD-Q pH sensor, shown in the inset of Figure 2b, in buffer solutions of pH 4, 7, and 9. The SWV peak observed is due to the 2e^–^2H^+^ PCET reaction occurring at the Q sites integrated into the sp^2^ carbon surface in the BDD electrode. Figure 2b shows the shift in the PCET peak potential, *E*_pH_, with solution pH. A slope of −56 mV/pH unit is observed, very close to theory (−58 mV/pH for a temperature of 21 °C) with an *R* ^2^ value of 0.9993.

**Figure 2:**
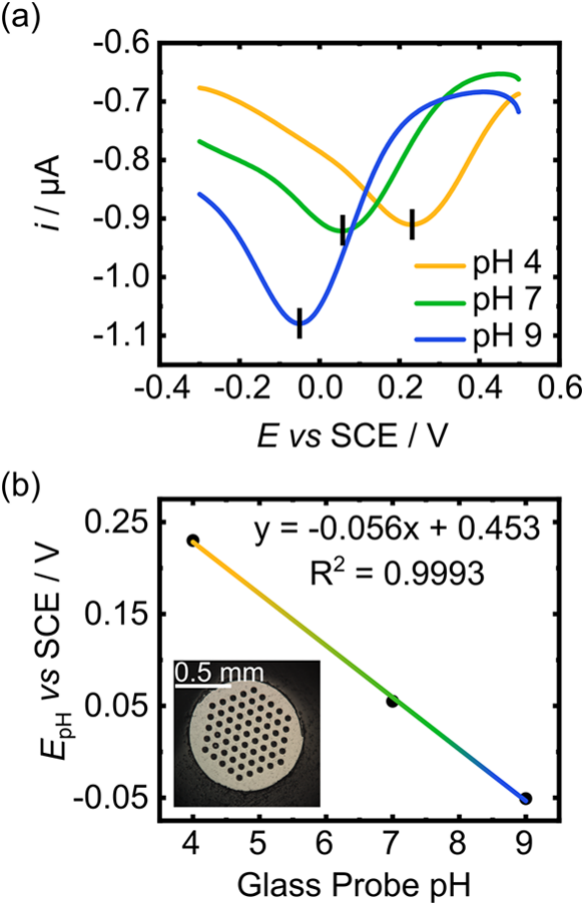
(a) SWV scans (100 Hz frequency, 0.001 V increment, 0.1 V amplitude, 2 s quiet time) for 2e^–^2H^+^ PCET at a BDD-Q electrode (scanning from 0.5 to −0.3 V vs SCE) in buffer solutions of pH 4 (yellow), 7 (green), and 9 (blue). (b) Resulting calibration curve of *E*_pH_ versus solution pH (measured by using a glass pH probe). The inset shows an optical image of the BDD-Q electrode; the dark circular regions represent the laser machined sp^2^ carbon regions.

There are five key requirements for an IREF redox species for any voltammetric SWV (or CV) pH measurement. (1) The IREF SWV response shows no dependence on pH over the pH range of interest. (2) IREF is chemically stable in the solution of interest for the time period required. (3) The SWV peak is sufficiently separated from the PCET pH peak, such that the two can be easily resolved, with (4) no peak interactions, due to the proximity of the two peaks, which result in an apparent shift of either peak from their true peak positions. (5) The redox species undergoes fast electron transfer, which results in narrower SWV peaks,^41^ with no follow up reactions. Additional requirements reflect the chemistry of the BDD voltammetric pH sensor. Specifically, there are no interactions (e.g., chemical or redox) between the redox species and surface integrated quinones of the BDD electrode, which detrimentally impact the IREF peak position.

Three fast electron transfer redox species, FcTMA^+^, Fe­(phen)_3_^2+^, and IrCl_6_^2–^, were chosen for the investigation,^100,35,42^ with formal electrode potentials of 0.392, 0.861, and 0.695 V vs SCE, respectively (Figure S1, SI-2), which lie outside the *E*_pH_ range, −0.05 V to +0.23 V vs SCE (Figure 2a). SWVs for the reduction of 100 μM (a) FcTMA^+^, (b) Fe­(phen)_3_^2+^, and (c) IrCl_6_^2–^ at a 1 mm diameter BDD-Q pH electrode in buffered solutions of pH 4, 7, and 9 are shown in Figure 3a–c.For SWV, given the potential is continuously switched between forward and backward pulses,^43^ we can choose to start scanning from either the positive or negative potential direction. The decision to scan in the negative potential direction was made to allow scope for scanning further in the negative direction, if required for future experiments, for example, to simultaneously voltammetrically measure dissolved oxygen, as well as pH/dissolved CO_2_.^30^

**Figure 3:**
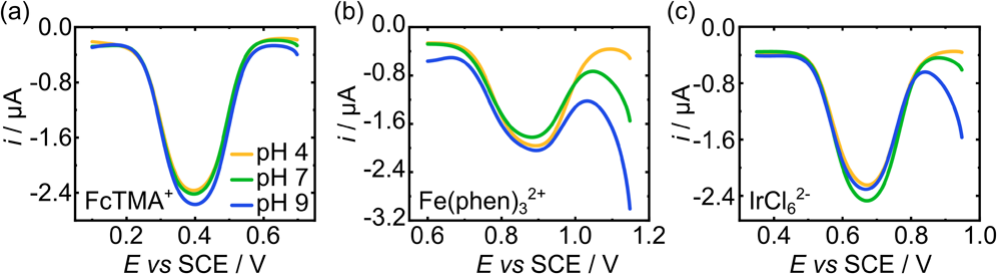
SWV scans (parameters as in Figure 2) on a BDD-Q electrode in buffer solutions of pH 4 (yellow), 7 (green), and 9 (blue) in the presence of 100 μM (a) FcTMA^+^ (scanning from 0.7 to 0.1 V vs SCE), (b) Fe­(phen)_3_^2+^ (1.15–0.6 V vs SCE), and (c) IrCl_6_^2–^ (0.85–0.35 V vs SCE).

All three IREF redox species showed good peak potential stability over a range of 5 pH units (pH 4–9); FcTMA^+^, Fe­(phen)_3_^2+^, and IrCl_6_^2–^ varied in *E*_IREF_ (peak potential of the internal reference species) by only 3, 9, and 5 mV, respectively, over the three measurements (when considering the maximum difference in *E*_IREF_ between the three SWVs). This corresponds to a maximum error of 0.05, 0.15, and 0.08 pH units (assuming a theoretical −59 mV/pH calibration at 298 K). The peak shape of the SWV is controlled by the SWV parameters employed (see Experimental Section).^41^ *E*_IREF_ values versus pH for the three IREF redox species are displayed in Table S2, SI-3.

For FcTMA^+^, despite displaying the smallest *E*_IREF_ shift, at a concentration of 100 μM, when scanning from a more positive start potential to reveal the pH peak, the separation between *E*_pH_ and *E*_IREF_ is not sufficient to resolve the pH peak for pH values ≤ 7, as shown in Figure S2a, SI-4. Whilst decreasing the FcTMA^+^ concentration (to 40 μM, Figure S2b) results in the pH peak at pH 7 being resolved, the pH 4 peak is still obscured by that associated with IREF, eliminating FcTMA^+^ as an IREF candidate over this pH range. Moreover, even if both peaks are prominent, depending on their separation and relative peak heights with respect to each other, the experimentally measured *E*_pH_ and *E*_IREF_ values may no longer be reflective of the actual *E*_pH_ and *E*_IREF_ values.

This concept is illustrated in Figure S3, SI-5, where for mathematical modeling simplicity the two peaks are treated as Gaussian in shape, with a peak width at half height of 200 mV (the latter reflecting the data in Figure 3a, where the peak width at half height is 200 mV for FcTMA^+^). The peaks going from left to right represent the pH and IREF peaks, respectively, as in real experimental data. The IREF peak is defined with an *E*_IREF_ of 0.4 V. The data is plotted for peak current (amplitude) ratios of pH:IREF of 1.0, 0.5, and 0.25 (the latter reflective of experimental data) and peak-to-peak separations of 0.2, 0.25, 0.3, and 0.34 V. A 0.34 V separation between the two peaks is shown to be the minimum separation required for the two peaks to be considered non-interacting, irrespective of peak current ratio. As the peak separation is decreased, the apparent peaks shift toward each other with respect to their actual positions. This results in an error in the measured values for *E*_IREF_, *E*_pH_, and *E*_diff_ and is exacerbated for the smaller peak, especially when the ratio of peak current pH:IREF is decreased. When Gaussian shaped peaks and a minimum peak separation of 0.34 V between *E*_IREF_ and the most positive *E*_pH_ value (at pH 4) is assumed, there is a requirement for IREF to have an *E*_IREF_ > 0.57 V vs SCE. Note, whilst our focus here is on apparent shifts in peak position on the voltage axis, peak separations should also be taken into account when analyzing closely spaced peak currents in multiple analyte measurements.^44^ The impact of peak seaparation on reporting the true peak current is shown in Table S3, SI-5.

The position of the IREF peak with respect to the commencement of water oxidation was also considered. When the solution pH increases, the potential at which oxidation of water begins becomes less positive. Figure 3b shows the impact of water oxidation on the SWV for Fe­(phen)_3_^2+^, which has the most positive *E*_IREF_ of the three species. As the pH is increased from 4 to 7 and 9, the SWV response for water oxidation becomes more noticeable. At pH 10, there is no peak evident in the SWV for Fe­(phen)_3_^2+^ due to its proximity to water oxidation (Figure S4, SI-6). Whilst the IREF peak is still visible at pH 9, the close spacing of the two responses has resulted in a small positive apparent shift of *E*_IREF_. Therefore, despite no convolution with the pH peak (data not shown), this couple is unusable at the higher pH values. Furthermore, given that water oxidation results in a local decrease in pH, in unbuffered solutions, due to proton generation, it is always advisable to minimize current flow arising from water oxidation during a voltammetric measurement of solution pH. Taking into account the proximity of the IREF SWV to the SWV response for water oxidation and the pH peak(s), for pH 4–9, for a BDD-Q electrode, this sets *E*_IREF_ to within 0.57–0.70 V vs SCE.

Figure 3c shows the SWV response for 100 μM IrCl_6_^2–^ over the pH range of 4–9; *E*_IREF_ is 0.670 V vs SCE, which sits at sufficient distances from both the pH and water oxidation SWVs such that the peaks can be treated as non-interacting. The response for water oxidation becomes increasingly visible as the pH increases. As highlighted above, the pH error is ∼0.08 pH due to the maximum 5 mV *E*_IREF_ variation. This is further reduced to 1 mV (∼0.02 pH error) for a pH of 6–8 (Table S2, SI-3). This latter range is appropriate for dissolved CO_2_ measurements in blood. This redox species was, therefore, used for all further measurements. Whilst these variations are smaller than all reports in the literature to-date, additional reasons for variations in *E*_IREF_ are currently the subject of further investigation.

To test the suitability of IrCl_6_^2–^ as an internal reference and the use of *E*_diff_ as a measurement signal, initial experiments used a standard reference electrode (SCE) and a silver chloride coated Ag foil (Ag|AgCl) RE in three different buffer solutions of pH 4, 6, and 8. The Cl^‑^ concentration was kept fixed for the Ag|AgCl RE experiments at 0.1 M, resulting in an *E*_Ag|AgCl_ of +0.049 V vs SCE (at 21 °C). A BDD-Q electrode was used to measure the solution pH via SWV referenced against (i) a SCE (black squares); (ii) a SCE in the presence of 100 μM IrCl_6_^2–^ (red circles), and (iii) a Ag|AgCl|Cl^–^ (0.1 M) RE in the presence of 100 μM IrCl_6_^2–^ (blue triangles), Figure 4. The SWVs recorded in the presence of IrCl_6_^2–^ show both pH and IREF peaks, allowing *E*_diff_ to be calculated. Figure 4 has two *y* axes, *E*_pH_ (no IrCl_6_^2–^) and *E*_diff_ (IrCl_6_^2–^ present). As can be seen from the linear regression values, while the intercepts may be different, a negligible difference in linearity and slope gradient is observed whether using *E*_diff_ or *E*_pH_ (*R* ^2^ values >0.999). Both of the calibration lines, which use *E*_diff_, fall within the 95% confidence interval (CI) of the BDD-Q SWV SCE calibration line.

**Figure 4:**
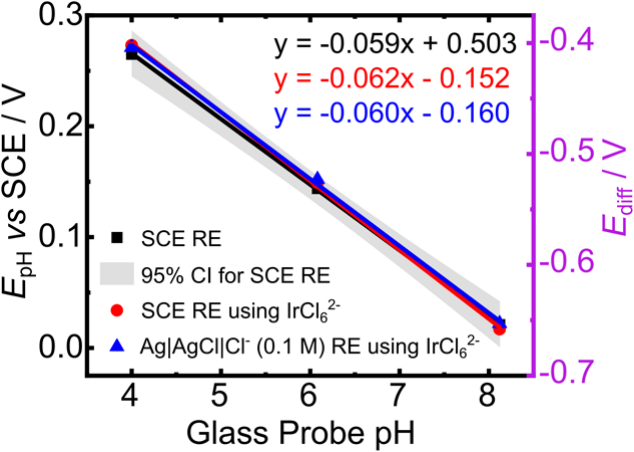
Buffered pH calibration for the BDD-Q electrode (SWV parameters as in Figure 2). On the left, *E*_pH_ is plotted with (i) SCE as the reference electrode (black squares) with a 95% CI (grey band). On the right, *E*_diff_ is plotted with (ii) IrCl_6_^2–^ as the IREF vs SCE (red circle) and (iii) IrCl_6_^2–^ as the IREF vs a Ag|AgCl|Cl^–^ (0.1 M) RE (blue triangle).

In order to assess the ability of the IrCl_6_^2–^ IREF system to account for RE drift, AgCl coated Ag foil was again employed as the RE and the concentration of Cl^–^ deliberately varied from 0.01 to 0.1 M to cause a quantifiable shift in the RE potential. For these experiments, the total salt concentration was kept at 0.1 M via additions of KNO_3_ (SI-7), removing any potential influence from ohmic drop. In practical applications, RE drift is very unlikely to be associated with a significant decrease in solution conductivity. Figure 5a,b shows SWV scans for a BDD-Q electrode in three pH 5 Carmody buffer solutions containing 0.01, 0.03, and 0.1 M KCl, with all solutions containing 100 μM IrCl_6_^2–^. In (a), a commercial SCE is used as the RE, while in (b), a Ag|AgCl foil RE is employed. As shown in Figure 5c, when measuring *E*_IREF_ vs SCE (black squares) for IrCl_6_^2–^ in the three different chloride concentration solutions, there is very little change in potential (3 mV). In contrast, *E*_IREF_ shifts by 53 mV when measured using the Ag|AgCl foil RE (black circles), due to the potential determining Cl^–^ ion concentration changes. In accordance with the Nernst equation, a change in Cl^–^ concentration from 0.01 to 0.1 M is theoretically expected to result in a potential change at the Ag|AgCl RE of 58 mV (at 21 °C), close to that observed here. *E*_diff_ values for the three different Cl^–^ solutions (purple triangles) were found to be almost identical as expected in a pH buffered solution: −496 mV (0.01 M), −495 mV (0.03 M), and −494 mV (0.1 M), highlighting the ability of the *E*_diff_ measurement to accommodate a shift in RE potential. The *E*_diff_ values can be converted to pH via the calibration line shown in Figure S5, SI-8, giving pH values of 4.97 (0.01 M), 4.96 (0.03 M), and 4.94 (0.1 M).

**Figure 5:**
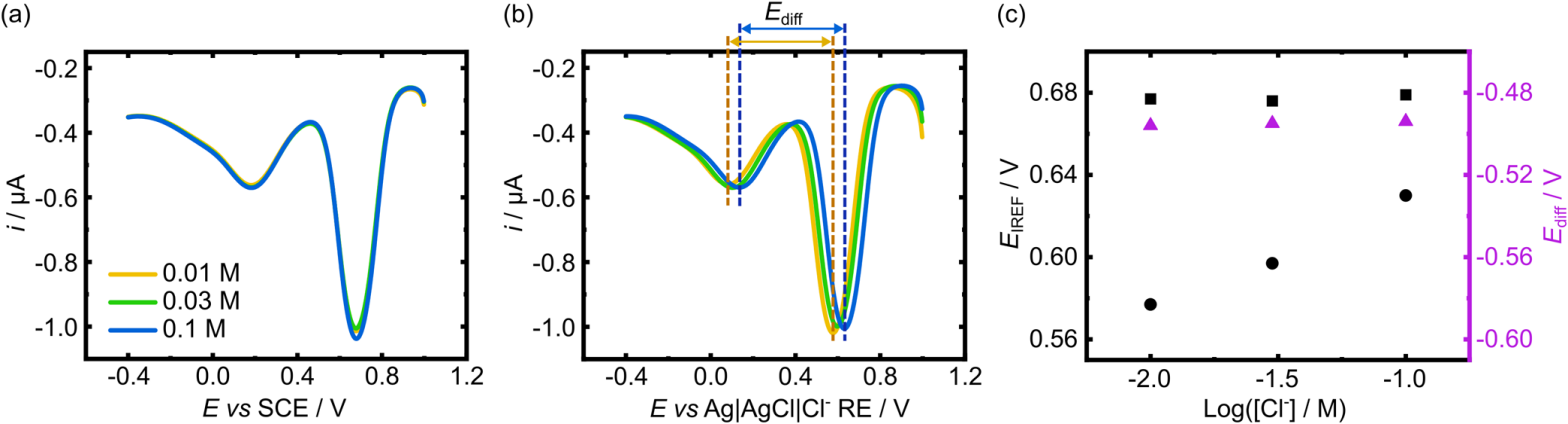
SWV (parameters as in Figure 2, scanning from 1.0 to −0.4 V) of pH and IREF (IrCl_6_^2–^) in KCl concentrations varying from 0.01–0.1 M, using (a) a SCE, and (b) a Ag|AgCl foil RE. (c) Plot of (left) *E*_IREF_ for a SCE RE (black squares) and Ag|AgCl foil RE (black circles); (right) *E*_diff_ versus log_10_ Cl^–^ concentration (purple triangles) for the data from (b).

Further proof-of-concept experiments were carried out using the BDD-Q electrode in a Stow-Severinghaus dissolved CO_2_ sensor arrangement,^10–12^ to verify whether *E*_diff_ could be used instead of *E*_pH_ as the SWV measurement signal. For these measurements, 100 μM IrCl_6_^2–^ was added to a solution containing 20 mM KHCO_3_ and 0.2 M KCl. Different partial pressures of CO_2_ (pCO_2_) in the range of 30.4–152.0 mmHg (corresponding to 4–20% CO_2_ of the total gas flow) were achieved by changing the composition of CO_2_:Ar flowing into the solution (SI-9). As the % of CO_2_ in the gas bubbled through the solution increases a buffer system is established (albeit weak given the concentrations of dissolved CO_2_ and HCO_3_^–^ involved). The dissolved CO_2_–pH relationship can be described using a modified form of the Henderson-Hasselbalch equation (eq 2), where, 0.034 is the Henry’s Law constant for dissolved CO_2_, in mmol L^–1^ mmHg^–1^ ^45^ and

$$\mathrm{pH}={\mathrm{pK}}_{a}+\log\left(\frac{\left[{{\mathrm{HCO}}_{3}}^{-}\right]}{0.034\times {\mathrm{pCO}}_{2}}\right)$$ 2

pK_a_ is the acid dissociation constant, equal to 6.3.^46^ Equation 2 can be simplified to a log–linear relationship when a known HCO_3_^–^ concentration is added to the electrolyte solution. In this case, 20 mM KHCO_3_ is used, giving eq 3, which predicts that an increase in pCO_2_ is associated with a decrease in pH.

$$\mathrm{pH}=-\log\left({\mathrm{pCO}}_{2}\right)+9.06$$ 3

Figure 6a shows BDD-Q SWVs recorded in a solution containing 20 mM HCO_3_^–^, 200 mM KCl, and 100 μM IrCl_6_^2–^ (IREF) for different pCO_2_ values in the range of 30.4 to 152 mmHg. As the pCO_2_ levels increase, the pH peak can be seen to shift to the right (by 40 mV) as expected for a solution that is decreasing in pH. Also shown is the SWV peak associated with 100 μM IrCl_6_^2–^. *E*_IREF_ changes by no more than 4 mV (a change of 0.07 pH units or log­(pCO_2_) units) over the course of the experiment.

**Figure 6:**
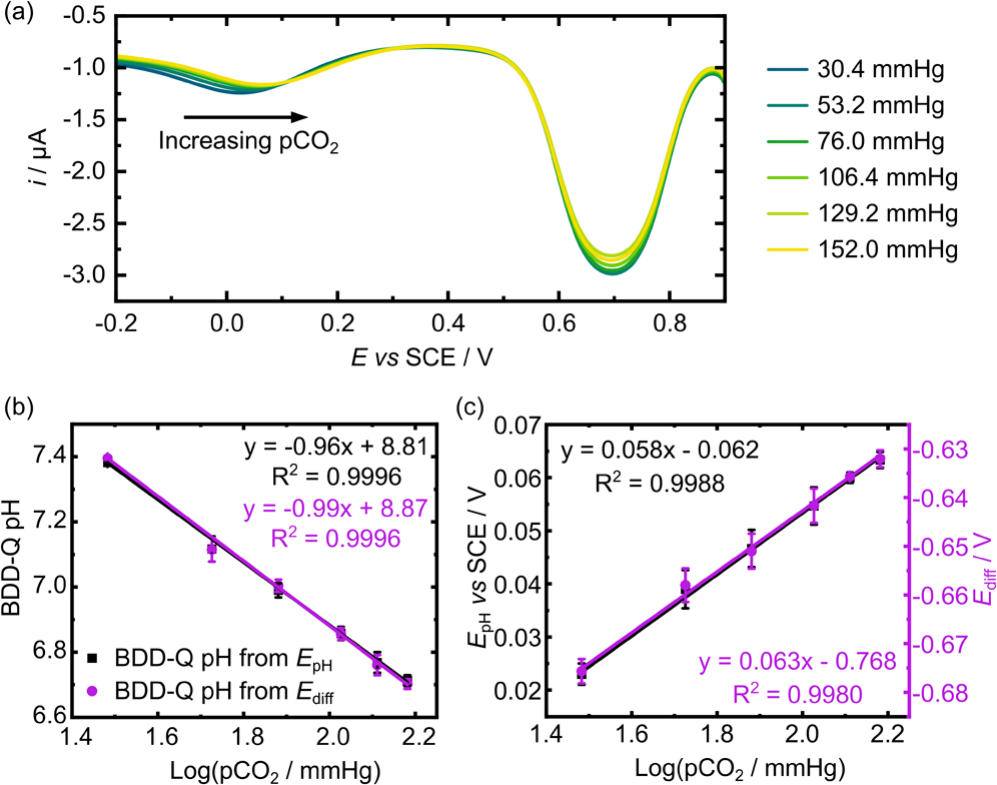
(a) SWV data (parameters as in Figure 2, scanning from 0.9 to −0.2 V vs SCE) showing the BDD-Q SWV pH peak and the IREF IrCl_6_^2–^ peak in one voltammetric scan. The arrow indicates the movement of the pH peak as pCO_2_ increases. (b) BDD-Q pH vs change in pCO_2_ calculated using calibration lines for *E*_pH_ (black squares) and *E*_diff_ (purple circles), the latter using 100 μM IrCl_6_^2–^ as the IREF. (c) Calibration plot of *E*_pH_ (left *y*-axis, black squares) and *E*_diff_ (right *y*-axis, purple circles) versus log­(pCO_2_). For (b) and (c), *n* = 3 where measurements were made using the same BDD-Q electrode in fresh solutions.

In Figure 6b, the resulting BDD-Q pH values, calculated from calibration plots using *E*_pH_, i.e., without IrCl_6_^2–^ (black squares) and *E*_diff_, i.e., with (purple circles) IrCl_6_^2–^ IREF, are plotted vs log­(pCO_2_); the pH varies from 6.7 to 7.4. The corresponding *E*_pH_ and *E*_diff_ calibration plots are shown in SI-10 and are recorded in the same bicarbonate electrolyte system used for Figure 6. Calibration line 1 plots BDD-Q *E*_pH_ versus the glass probe pH, resulting in a calibration slope of −56 mV/pH unit (*R* ^2^ = 0.9995), Figure S6a, SI-10. Calibration line 2 (which uses IREF) plots *E*_diff_ versus the glass probe pH, resulting in a calibration slope of −61 mV/pH unit (*R* ^2^ = 0.9985), Figure S6b, SI-10. The gradients of the two plots in Figure 6b are very close to −1 pH unit per mmHg pCO_2_, with a *y*-intercept just below the expected 9.06 pH units (*R* ^2^ > 0.999 for both sets of data, *n* = 3). Hence, the BDD-Q CO_2_ sensor follows the predicted response (eq 3) for both scenarios where either a standard reference (*E*_pH_) or IREF (*E*_diff_) is utilized. Figure 6c shows plots of *E*_pH_ (black squares) and *E*_diff_ (purple circles) against log­(pCO_2_), which is a simpler form of calibration, as it does not require conversion of either potential to a pH value first. For both, a linear line is expected with a Nernstian slope of 59 mV/log­(pCO_2_) unit (at 298 K), derived as shown in SI-11. Slopes very close to these values of 58 mV/log­(pCO_2_) (*E*_pH_) and 63 mV/log­(pCO_2_) (*E*_diff_) are obtained, with *R* ^2^ > 0.99 and *n* = 3. Figure 6b,c highlights the validity of the IREF (*E*_diff_) approach for measuring the dissolved CO_2_.

## Conclusion

For suitability as an internal reference redox species for voltammetric pH determination, IREF must display a SWV response sufficiently removed from the pH and water electrolysis responses such that they can be considered non-interacting. If present, apparent shifts in IREF/pH peak position are seen, resulting in errors in measurement of *E*_pH_, *E*_IREF_, and *E*_diff_. Whilst this concept applies universally, for the BDD-Q pH electrode, *E*_IREF_ was estimated as needing to fall within the range of 0.57–0.70 V vs SCE for pH 4–9. IrCl_6_^2–^ was found to be the most promising IREF for this pH measurement system, showing a maximum variation in SWV peak potential of only 5 mV over a five orders of magnitude change in proton concentration, equivalent to a pH error of ∼0.08. Reducing the pH range to that of importance in medical applications, pH = 6–8, resulted in a pH error of ∼0.02. Current applications would be determined by the level of pH accuracy required. The study was extended to show, for the first time, the versatility of the *E*_diff_ method in measuring dissolved CO_2_ concentrations via the pH response of a weakly buffered bicarbonate solution.

Currently, IREF is added to the solution and is thus appropriate for sampled solutions analyzed in a laboratory or for sensors where the electrolyte solution is separated from the analytical solution by a solution-impermeable membrane. For making membraneless measurements at the source, e.g., directly in an environmental sample, integration of IREF into the surface of the electrode, via surface grating or drop casting of a film, is required. Furthermore, while the variation in IREF peak potential as a function of pH is at best 1 mV over pH 6–8 (at worst 5 mV, over pH 4–9), there is still scope for further improvements. Although IrCl_6_^2–^ has shown promise, it is more expensive compared to the Fe-based redox couples. Thus, there is the option for producing cheaper metal centered redox species where the functional groups attached to the metal center have been tuned to result in redox potentials that fall within the window of interest, here 0.57–0.70 V vs SCE.

There is also a need to explore the impact of elevated temperatures or significant use time scales. Both are of importance for dissolved CO_2_ Stow-Severinghaus transcutaneous sensors where the electrolyte is heated to 43 °C during extended use on a patient.^47^ These measurements typically run continuously for up to 1 week at a time. Continuous long-term monitoring is where we see the IREF methodology offering most advantages, due to the greater possibility of RE drift, and is where future work is directed.

The concepts discussed here with respect to ensuring there are no peak interactions can be extended to other voltammetric pH sensors used in conjunction with IREF and alternative sensing methods. For the latter, this includes, for example, where the peak current is related to analyte concentration for multiple analyte detection using the same sensor. Finally, it is also possible to consider data processing, post-collection, to deconvolute closely spaced and overlapping peaks. This would extend the workable potential range for the IREF and enable a wider pH range to be accessed.

## Supplementary Material



## Notes

1: MATLAB Code; 2: Cyclic Voltammetry of Reversible Redox Species on BDD-Q Electrodes; 3: pH Stability of Redox Species; 4: SWV FcTMA^+^ Overlap with BDD-Q pH peak; 5: Mathematically Determining Required Peak Separation; 6: 100 μM Fe­(phen)_3_^2+^ in pH buffers 4, 7, 9 and 10; 7: Conductivity of Solutions Used for Drifting Reference Experiments; 8: *E*_diff_ vs pH Calibration for Changing [Cl^–^] Experiment; 9: Carbon Dioxide and Argon Compositions for CO_2_ Experiment; 10: CO_2_ Calibration Curves; 11: Potential vs log­(pCO_2_) Theory (PDF)

## References

[1] Bard, A. J. ; Faulkner, L. R. ; White, H. S. Electrochemical Methods: Fundamentals and Applications, 3rd ed.; Wiley, 2023.

[2] Shinwari M. W., Zhitomirsky D., Deen I. A., Selvaganapathy P. R., Deen M. J., Landheer D. (2010). Microfabricated Reference Electrodes
and Their Biosensing *Applications*. Sensors.

[3] Lammel C., Heubner C., Liebmann T., Schneider M. (2017). Critical Impact
of Chloride Containing Reference Electrodes on Electrochemical Measurements. Electroanalysis.

[4] Brezinski D. P. (1982). Use of
Half-Cell Barriers to Eliminate Junction Clogging and Thermal Hysteresis
in Silver/Silver Chloride Reference Electrodes. Anal Chim Acta.

[5] Macpherson J. V., Unwin P. R. (1996). Scanning Electrochemical Microscope-Induced
Dissolution:
Theory and Experiment for Silver Chloride Dissolution Kinetics in
Aqueous Solution without Supporting Electrolyte. J. Phys. Chem..

[6] Bentley C. L., Perry D., Unwin P. R. (2018). Stability
and Placement of Ag/AgCl
Quasi-Reference Counter Electrodes in Confined Electrochemical Cells. Anal Chem..

[7] Westcott, C. C. pH Measurements; Elsevier, 1978; 10.1016/B978-0-12-745150-3.X5001-2.

[8] Głáb S., Hulanicki A., Edwall G., Ingman I. (1989). Metal-Metal Oxide and
Metal Oxide Electrodes as pH Sensors. Crit Rev.
Anal Chem..

[9] Ayres Z. J., Borrill A. J., Newland J. C., Newton M. E., Macpherson J. V. (2016). Controlled
sp^2^ Functionalization of Boron Doped Diamond as a Route
for the Fabrication of Robust and Nernstian pH Electrodes. Anal Chem..

[10] Severinghaus J. W., Bradley F. (1958). Electrodes for Blood pO_2_ and pCO_2_ Determination. J. Appl.
Physiol..

[11] Delost, M. Blood Gas and Critical Care Analyte Analysis. In Equipment for Respiratory Care; 2014; pp 151–174.

[12] Severinghaus, J. W. CO_2_ Electrodes. In Encyclopedia of Medical Devices and Instrumentation; 2006; 10.1002/0471732877.emd326.

[13] Zosel J., Oelßner W., Decker M., Gerlach G., Guth U. (2011). The Measurement
of Dissolved and Gaseous Carbon Dioxide Concentration. Meas Sci. Technol..

[14] Restrepo R. D., Hirst K. R., Wittnebel L., Wettstein R. (2012). AARC Clinical
Practice Guideline: Transcutaneous Monitoring of Carbon Dioxide and
Oxygen: 2012. Respir Care.

[15] Rubinstein I. (1984). Voltammetric
pH Measurements with Surface-Modified Electrodes and a Voltammetric
Internal Reference. Anal Chem..

[16] Gagne R. R., Koval C. A., Lisensky G. C. (1980). Ferrocene as an Internal Standard
for Electrochemical Measurements. Inorg. Chem..

[17] Hickman J. J., Ofer D., Laibinis P. E., Whitesides G. M., Wrighton M. S. (1991). Molecular Self-Assembly of Two-Terminal, Voltammetric
Microsensors with Internal References. Science.

[18] Levey K. J., Macpherson J. V. (2024). A Current Averaging Strategy for Maximizing Analyte
and Minimizing Redox Interference Signals with Square Wave Voltammetry. Anal Chem..

[19] Lahav M., Katz E., Willner I. (1998). A Covalently
Linked Quinone-Ferrocene
Monolayer-Electrode: A pH Sensor with an Internal Reference. Electroanalysis.

[20] Lafitte V. G. H., Wang W., Yashina A. S., Lawrence N. S. (2008). Anthraquinone-Ferrocene
Film Electrodes: Utility in pH and Oxygen Sensing. Electrochem commun.

[21] Xiong L., Batchelor-Mcauley C., Compton R. G. (2011). Calibrationless pH Sensors Based
on Nitrosophenyl and Ferrocenyl Co-Modified Screen Printed Electrodes. Sens Actuators B Chem..

[22] Musa A. E., Alonso-Lomillo M. A., Del Campo F. J., Abramova N., Domínguez-Renedo O., Arcos-Martínez M. J., Kutter J. P. (2012). Thick-Film Voltammetric
pH-Sensors with Internal Indicator and Reference Species. Talanta.

[23] Bailey S. I., Ritchie I. M. (1985). A Cyclic Voltammetric Study of the
Aqueous Electrochemistry
of Some Quinones. Electrochim Acta.

[24] Sisodia N., Mcguinness K. L., Wadhawan J. D., Lawrence N. S. (2022). In Situ Recalibration
of Ion Selective Electrodes. Sensors & Diagnostics.

[25] Pöller S., Schuhmann W. (2014). A Miniaturized
Voltammetric pH Sensor Based on Optimized
Redox Polymers. Electrochim Acta.

[26] Cobb S. J., Laidlaw F. H. J., West G., Wood G., Newton M. E., Beanland R., Macpherson J. V. (2020). Assessment
of Acid and Thermal Oxidation
Treatments for Removing sp^2^ Bonded Carbon from the Surface
of Boron Doped Diamond. Carbon.

[27] Cobb S. J., Ayres Z. J., Newton M. E., Macpherson J. V. (2019). Deconvoluting
Surface-Bound Quinone Proton Coupled Electron Transfer in Unbuffered
Solutions: Toward a Universal Voltammetric pH Electrode. J. Am. Chem. Soc..

[30] Read T. L., Cobb S. J., Macpherson J. V. (2019). An sp^2^ Patterned Boron
Doped Diamond Electrode for the Simultaneous Detection of Dissolved
Oxygen and pH. ACS Sens.

[100] Petrovic S. (2000). Cyclic Voltammetry of Hexachloroiridate­(IV):
An Alternative
to the Electrochemical Study of the Ferricyanide Ion. Chem. Educator.

[31] Ostle, C. ; Williamson, P. ; Artioli, Y. ; Bakker, D. C. E. ; Birchenough, S. ; Davis, C. E. ; Dye, S. ; Edwards, M. ; Findlay, H. S. ; Greenwood, N. ; Hartman, S. ; Humphreys, M. P. ; Jickells, T. ; Johnson, M. ; Landschützer, P. ; Parker, R. ; Pearce, D. ; Pinnegar, J. ; Robinson, C. ; Schuster, U. ; Silburn, B. ; Thomas, R. ; Wakelin, S. ; Walsham, P. ; Watson, A. J. Carbon Dioxide and Ocean Acidification Observations in UK Waters: Synthesis Report with a Focus on 2010–2015; 2016; 10.13140/RG.2.1.4819.4164.

[32] Malyan S. K., Singh O., Kumar A., Anand G., Singh R., Singh S., Yu Z., Kumar J., Fagodiya R. K., Kumar A. (2022). Greenhouse Gases Trade-Off
from Ponds: An Overview of Emission Process
and Their Driving Factors. Water (Switzerland).

[33] Feher, J. Acid-Base Physiology I: The Bicarbonate Buffer System and Respiratory Compensation. In Quantitative Human Physiology, Second ed.; 2017; pp 665–671.

[34] Carmody W. R. (1961). An Easily
Prepared Wide Range Buffer Series. J. Chem.
Educ.

[35] Lemay S. G., Van Den Brook D. M., Storm A. J., Krapf D., Smeets R. M. M., Heering H. A., Dekker C. (2005). Lithographically Fabricated Nanopore-Based
Electrodes for Electrochemistry. Anal Chem..

[36] Hutton L.
A., Iacobini J. G., Bitziou E., Channon R. B., Newton M. E., Macpherson J. V. (2013). Examination
of the Factors Affecting the Electrochemical
Performance of Oxygen-Terminated Polycrystalline Boron-Doped Diamond
Electrodes. Anal Chem..

[37] Hutton L., Newton M. E., Unwin P. R., Macpherson J. V. (2009). Amperometric
Oxygen Sensor Based on a Platinum Nanoparticle-Modified Polycrystalline
Boron Doped Diamond Disk Electrode. Anal Chem..

[38] Daviddi E., Shkirskiy V., Kirkman P. M., Robin M. P., Bentley C. L., Unwin P. R. (2022). Screening
the Surface Structure-Dependent Action of
a Benzotriazole Derivative on Copper Electrochemistry in a Triple-Phase
Nanoscale Environment. J. Phys. Chem. C.

[39] Tobias J. D. (2009). Transcutaneous
Carbon Dioxide Monitoring in Infants and Children. Paediatr Anaesth.

[40] Cousineau J., Anctil S., Carceller A., Gonthier M., Delvin E. E. (2005). Neonate
Capillary Blood Gas Reference Values. Clin Biochem.

[41] Mirceski V., Skrzypek S., Stojanov L. (2018). Square-Wave
Voltammetry. ChemTexts.

[42] Martinez M. V., Coneo Rodriguez R., Baena Moncada A., Rivarola C. R., Bruno M. M., Miras M. C., Barbero C. A. (2016). Electrochemistry of Tris­(1,10-Phenanthroline)­Iron­(II)
inside a Polymeric Hydrogel. Coupled Chemical Reactions and Migration
Effects. J. Solid State Electrochem..

[43] Osteryoung J. G., Osteryoung R. A. (1985). Square Wave Voltammetry. Anal
Chem..

[44] Li X., Xia J., Li W., Zhang S. (2010). Multianalyte Electrochemical Biosensor
Based on Aptamer- and Nanoparticle-Integrated Bio-Barcode Amplification. Chem. Asian J..

[45] Sander R. (2015). Compilation
of Henry’s Law Constants (Version 4.0) for Water as Solvent. Atmos Chem. Phys..

[46] Burrows, A. ; Holman, J. ; Lancaster, S. ; Overton, T. ; Parsons, A. ; Pilling, G. ; Price, G. 7. Acids and Bases. In Chemistry3: Introducing Inorganic, Organic, and Physical Chemistry, Fourth ed.; Oxford University Press; 2021; pp 324–325.

[47] Janssens J. P., Perrin E., Bennani I., De Muralt B., Titelion V., Picaud C. (2001). Is Continuous Transcutaneous Monitoring
of *P*CO_2_ (Tc*P*CO_2_) over 8 h Reliable in Adults?. Respir Med..

