# Supplementary material for: Choosing the Correct Internal Reference Redox Species for Overcoming Reference Electrode Drift in Voltammetric pH Measurements
Source: ACS Electrochem. 2025 Jun 13;1(8):1532–9. doi: 10.1021/acselectrochem.5c00138 (PMC12337080; doi:10.1021/acselectrochem.5c00138)
Supplement: Supplementary file 1 [file ec5c00138_si_001.pdf]

Supporting Information

Choosing the Correct Internal Reference Redox Species for Overcoming Reference Electrode Drift in Voltammetric pH Measurements

Nafiz B. Biswas,[1] Tania Read,[1] Katherine J. Levey,[1,2] and Julie V. Macpherson[1,*]

[1]Department of Chemistry, University of Warwick, Coventry CV4 7AL

[2]Leiden Institute of Chemistry, Leiden University, Einsteinweg 55, 2333 CC Leiden, The Netherlands

*j.macpherson@warwick.ac.uk

Contents

SI-1: MATLAB Code 2

SI-1.1: Averaging and Smoothing of SWV Scans 2

SI-1.2: Finding the Peak Current Voltage Positions 3

SI-2: Cyclic Voltammetry of Reversible Redox Species on BDD-Q Electrodes 5

SI-3: pH Stability of Redox Species 6

SI-4: SWV $FcTMA^+$ Overlap with BDD-Q pH peak 7

SI-5: Mathematically Determining Required Peak Separation 8

SI-6: 100 µM $Fe(phen)_3^{2+}$ in pH buffers 4, 7, 9 and 10 10

SI-7: Conductivity of Solutions Used for Drifting Reference Experiments 11

SI-8: E_{diff} *vs* pH Calibration for Changing [Cl^-] Experiment 12

SI-9: Carbon Dioxide and Argon Compositions for CO_2 Experiment 13

SI-10: CO_2 Calibration Curves 14

SI-11: Potential *vs* log(pCO_2) Theory 15

References 16

SI-1: MATLAB Code

SI-1.1: Averaging and Smoothing of SWV Scans

```
% clear workspace
clear all; clc;

% format extracted data as ‘float’
formatSpec = '%f%f%f%f%f';

% put files you want in single folder and add to path
% select all files to process
[file,path] = uigetfile([pwd '\*.txt'],'MultiSelect','on');

% create folder (in current path) where averaged scans are placed
mkdir Averaged
finalpath = append(path,'Averaged\');

% Data Import
numfiles = length(file);
V_peak = zeros(numfiles,1);

% loop over total number of files (in this case each SWV has 6 repeats)
for k = 1:numfiles/6
    % loop for each set of 6 files to be averaged with first discarded
    for j = 2:6
        filename = char(file(1,((k-1)*6)+j));

        % find the startrow (e.g. containing 'potential')
        A = regexp(fileread(filename),'\n','split');

        % find the heading row of file
        startRow = find(contains(A,'Potential'));

        % open file
        fileID = fopen(filename);

        data = textscan(fileID, formatSpec, 'Delimiter',',',...
            'Headerlines',startRow+2);

        % close file
        fclose(fileID);

        % put file data into variables
        V = data{1};
        C{j} = data{2};
        sz = size(V);
    end

    % calculating average data and smoothing
    Voltage = V;
    C_Ave = (C{2}+C{3}+C{4}+C{5}+C{6})/5;
    C_AveSmooth = sgolayfilt(C_Ave,3,21);

    % name of output file
    name = convertStringsToChars(filename);
    finalname = char(append(name(1:end-5),'ave.txt'));

    % writing and saving of table with data of averaged scan
```

```
    table1 = table(Voltage,C_AveSmooth);
    writetable(table1,[finalpath finalname],'Delimiter','\t',...
        'WriteRowNames',true);
end
```

SI-1.2: Finding the Peak Current Voltage Positions

```
% clear workspace
clear all, clc;

% format extracted data as ‘float’
formatSpec = '%f%f%f%f';

% put files you want in single folder and add to path
% select all files to process
[file,path] = uigetfile([pwd '\*.txt'],'MultiSelect','on');

% find number of files being processed
numfiles = length(file);

% initialise variable arrays
pH_peak_final = zeros(numfiles,1);
IrCl_peak_final = zeros(numfiles,1);
pH_Values = zeros(numfiles,1);

% data point between the two peaks being determined (requires user input)
div = 100;

% loop over total number of files
for j = 1:numfiles
    filename = char(file(j));

    % open file
    fileID = fopen(filename);

    data = textscan(fileID, formatSpec, 'Delimiter','\t','Headerlines',1);

    % close file
    fclose(fileID);

    % put file data into variables
    V = data{1};
    C{j} = data{2};
    sz = size(V);

    % change variable from cell array to double array
    C1 = C{j};

    % find E_pH
    pH_peak = abs(C1(div));
    for i = div:sz
        if abs(C1(i)) >= pH_peak
            pH_peak = abs(C1(i));
            idx_1 = i;
        end
    end
    pH_peak_final(j) = V(idx_1);
```

```
    % find E_IrCl
    IrCl_peak = abs(C1(1));
    for i = 1:div
        if abs(C1(i)) >= IrCl_peak
            IrCl_peak = abs(C1(i));
            idx_2 = i;
        end
    end
    IrCl_peak_final(j) = V(idx_2);

    % find pH values
    pH_temp = extractBetween(filename,'_pH','_ave');
    pH_Values(j)=str2double(strrep(pH_temp,',','.'));

end

% output lists of E_ pH, E_IrCl, and pH Values
pH_peak_final
IrCl_peak_final
pH_Values
```

If using MacOS, path names will use '/' instead of '\' (e.g. in line 8 of the code). Some other MATLAB syntax might also differ.

SI-2: Cyclic Voltammetry of Reversible Redox Species on BDD-Q Electrodes

The reversibility of the one electron transfer redox couples, $FcTMA^{+}$, $Fe(phen)_3^{2+}$, and $IrCl_6^{2-}$ was investigated using cyclic voltammetry (CV), **Figure S1**. The peak to peak separation, ΔE_p, of a reversible (diffusion controlled) redox species should be 57/*n* mV, where *n* is the number of electrons transferred.[1] The ΔE_p values and formal potential, $E^{o'}$, values (mid-way position between the two peaks) are supplied in **Table S1**.

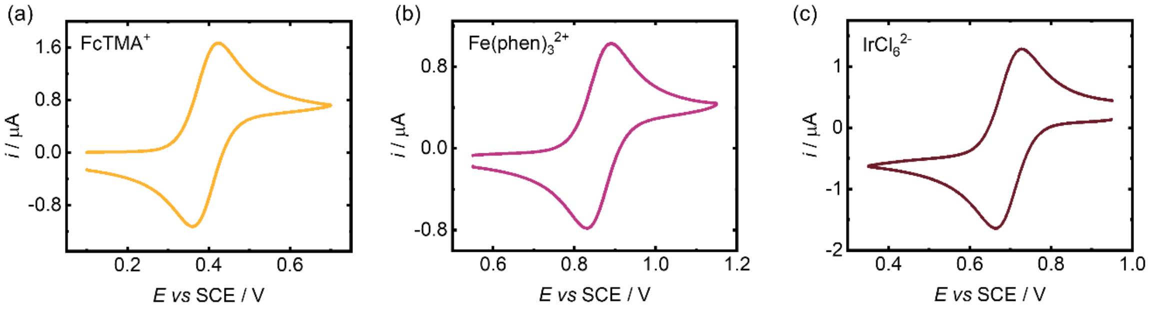

Figure S1. CV scans (1st scan) recorded using a BDD-Q electrode at 0.1 V s^{-1}, in pH 7 buffer containing (a) 1 mM $FcTMA^{+}$, (b) 1 mM $Fe(phen)_3^{2+}$, and (c) 1 mM $IrCl_6^{2-}$.

Table S1. Table showing ΔE_p and $E^{o'}$ values for $FcTMA^{+}$, $Fe(phen)_3^{2+}$, and $IrCl_6^{2-}$

Redox Species	ΔE_p / mV	$E^{o'}$ *vs* SCE / V
$FcTMA^{+}$	64	0.392
$Fe(phen)_3^{2+}$	59	0.861
$IrCl_6^{2-}$	64	0.695

SI-3: pH Stability of Redox Species

Table S2 shows the E_{IREF} *vs* SCE for pH values in the range 4 – 9. 100 μM of the redox species were added to standard Reagecon buffer solutions. The potential drift can be equated to a pH error, assuming 59 mV / pH, this leads to pH errors of 0.05 for $FcTMA^{+}$, 0.15 for $Fe(phen)_3^{2+}$, and 0.08 for $IrCl_6^{2-}$. When a pH range of 6 – 8 is used (relevant for dissolved CO_2 measurements in blood), the $IrCl_6^{2-}$ variation is reduced to 1 mV which equates to 0.02 pH.

Table S2. E_{IREF} values for $FcTMA^{+}$, $IrCl_6^{2-}$, and $Fe(phen)_3^{2+}$ at pH values ranging from 4 – 9.

pH	E_{IREF} *vs* SCE / V		
	100 μM $FcTMA^{+}$	100 μM $Fe(phen)_3^{2+}$	100 μM $IrCl_6^{2-}$
4	0.396	0.886	0.673
6			0.670
7	0.397	0.887	0.671
8			0.670
9	0.399	0.895	0.668

Ave.	0.397	0.889	0.670
Range	0.003	0.009	0.005
s.d.	0.001	0.004	0.002

SI-4: SWV $FcTMA^+$ Overlap with BDD-Q pH peak

For a $FcTMA^+$ concentration of 100 μM, it was found that the $FcTMA^+$ SWV peak (right) overlapped with the pH peak (left) for pH values ≤ 7, **Figure S2a**, making it impossible to identify E_{pH}. Lowering the $FcTMA^+$ concentration further, to 40 μM, revealed the pH 7 peak, **Figure S2b**, but the pH 4 peak was still unresolvable. The complete overlap and close spacing of the pH and IREF peaks results in apparent shifts of the $FcTMA^+$ peak position to less positive values as the pH decreases (E_{IREF} = 0.393, 0.386 and 0.379 V at pH 9, 7, and 4 respectively).

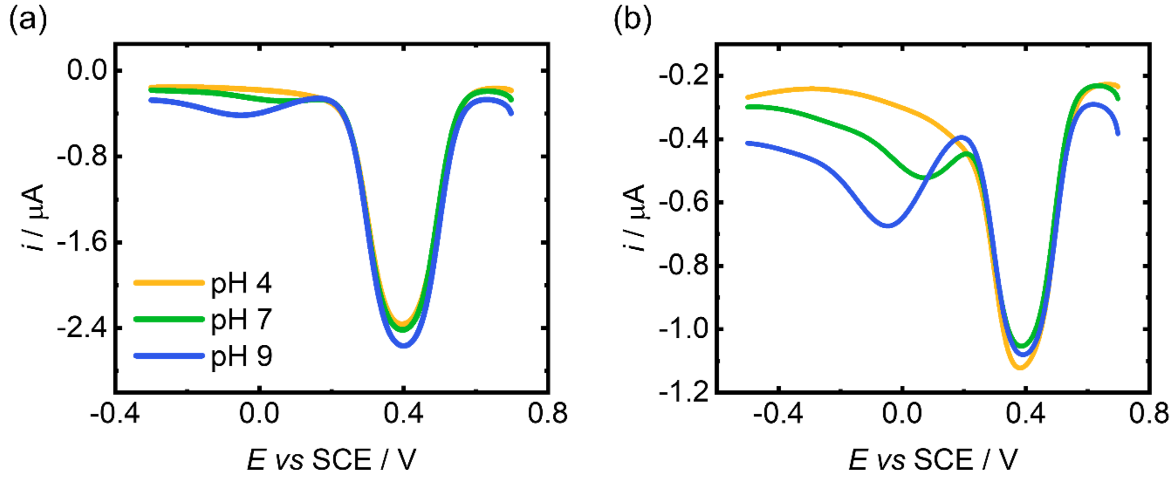

Figure S2. The pH SWV peak at pH 4, 7, and 9, with (a) 100 μM and (b) 40 μM $FcTMA^+$ (100 Hz frequency, 0.001 V increment, 0.1 V amplitude).

SI-5: Mathematically Determining Required Peak Separation

To illustrate the impact of closely spaced peaks, a MATLAB (R2021b, MathWorks®) model using Gaussian peaks was made. An offset of -0.2 µA from the 0 baseline was used to allow easier visualisation of peak shifts that occur as a result of peak summation.

In **Figure S3**, Peak 1 (E_{IREF}) was always set to 0.4 V with an amplitude of 2.2 µA and peak width at half height (FWHM) of 200 mV (mimicking the E_{IREF} for 100 µM $FcTMA^+$). Peak 2 (E_{pH}) was plotted with a varying current amplitude of (a) 2.2 µA (identical to Peak 1), (b) 1.1 µA (50% of Peak 1), and (c) 0.55 µA (25% of Peak 1). E_{pH} was set at (i) 0.06 V (0.34 V peak separation), (ii) 0.1 V (0.3 V separation), (iii) 0.15 V (0.25 V separation), and (iv) 0.2 V (0.2 V separation). The apparent shift of each peak is labelled on **Figure S3**. For equal amplitude peaks, when the peaks are close enough for peak summation, as the peak-to-peak separation decreases the greater the apparent shift, as shown in **Figure S3a**. When the two peaks are not equal in amplitude, the smaller peak undergoes greater apparent shifts in position which is exacerbated as the ratio of small peak amplitude to large peak amplitude decreases (**Figure S3b and c**).

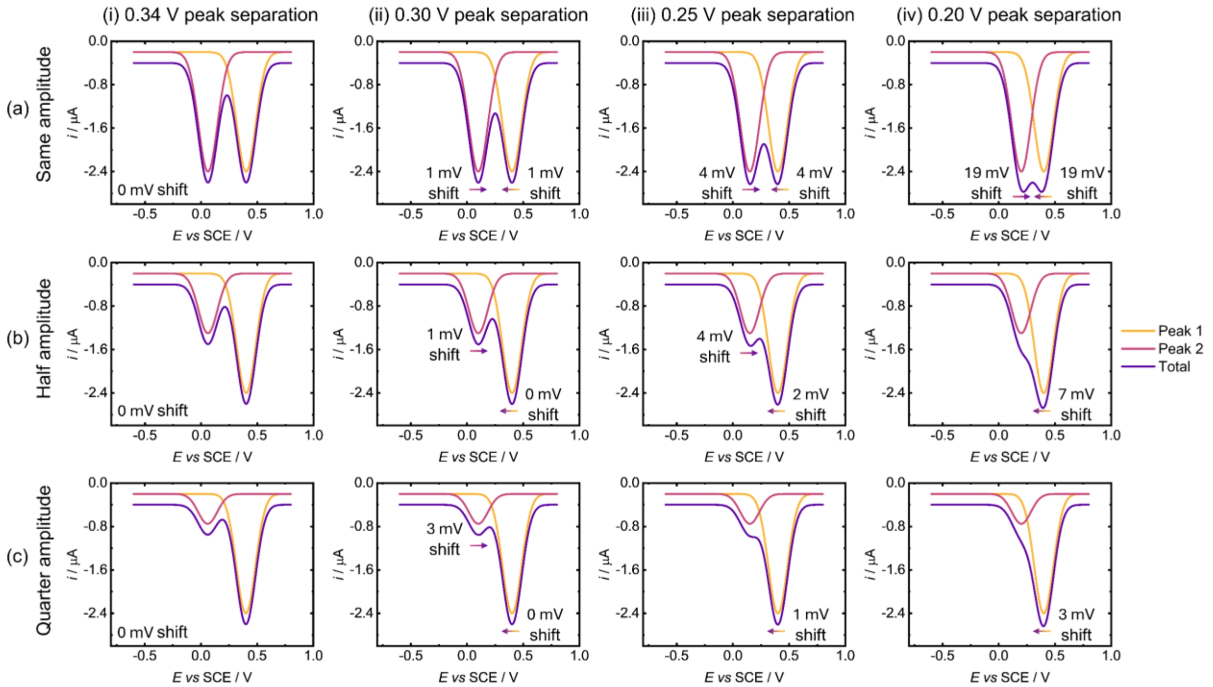

Figure S3. The model results for (i) 0.34 V, (ii) 0.3 V, (iii) 0.25 V and (iv) 0.2 V peak-to-peak separations for ratios of peak amplitude (pH) : peak amplitude (IREF) of (a) 1.0 (b) 0.5 and (c) 0.25.

As pH decreases the position of E_{pH} becomes more positive. At the lowest pH investigated, pH 4, E_{pH} ~ 0.23 V *vs* SCE, and the pH peak amplitude is ~ 25% that of the amplitude of the IREF peak at 100 μM concentration. Under these conditions, ~0.34 V is shown in Figure S3c to be the minimum peak-to-peak separation for no peak interactions. This dictates that E_{IREF} of an ideal IREF species for the BDD-Q electrode operating over the pH range 4-9, is > 0.57 V *vs* SCE. Note, similar concepts are used in chromatography, however here baseline resolution between adjacent peaks is required, which results in the need for bigger peak separations.

Table S3. Table summarising the percentage decrease in apparent peak current (compared to real peal current) for the data in **Figure S3**. NP = No observable peak

Peak Separation / V	Peak Position / V	% Change in Peak Current		
		Same Amplitude	½ Amplitude for Peak 2 compared to Peak 1	¼ Amplitude for Peak 2 compared to Peak 1
0.34	0.06	0.05	0.1	0.2
	0.40	0.00	0.00	0.00
0.30	0.10	0.2	0.4	0.9
	0.40	0.2	0.1	0.05
0.25	0.15	1.4	3.0	NP
	0.40	1.4	6.2	0.3
0.20	0.20	7.9	NP	NP
	0.40	7.9	3.5	1.6

SI-6: 100 µM $Fe(phen)_3^{2+}$ in pH buffers 4, 7, 9 and 10

Figure S4 shows the SWV response for $Fe(phen)_3^{2+}$ as a function of pH for 4, 7, 9 and 10. As the pH increases the $Fe(phen)_3^{2+}$ SWV response overlaps more with water oxidation, moving the peak position, E_{IREF}, slightly more positive (E_{IREF} = 0.886, 0.887, 0.895 *vs* SCE for pH 4,7, and 9 respectively), until the peak becomes unresolvable at pH ≥ 10.

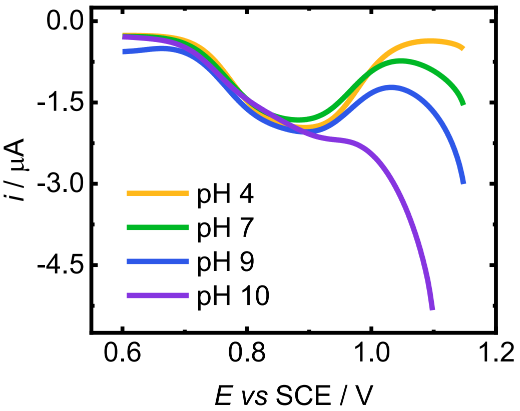

Figure S4. SWV (100 Hz frequency, 0.001 V increment, 0.1 V amplitude) of 100 µM $Fe(phen)_3^{2+}$ in pH buffer 4, 7, 9, and 10.

SI-7: Conductivity of Solutions Used for Drifting Reference Experiments

Conductivity measurements were taken using a Mettler Toledo InLab® 738-ISM conductivity probe in solutions with varying concentrations of KCl and KNO_3 (**Table S4**). By keeping the total salt concentration at 0.1 M, the conductivity of the solutions are kept within the same order of magnitude, with a range of 12.24 – 13.25 mS. This ensures that no ohmic drop effects are present while still allowing the chloride concentration to be altered.

Table S4. Conductivity of the solutions used in the drifting reference experiment

KCl Concentration / M	KNO_3 Concentration / M	Conductivity / mS
0.01	0.09	12.61
0.03	0.07	12.24
0.1	0	13.25

SI-8: E_{diff} *vs* pH Calibration for Changing [Cl^-] Experiment

Figure S5 shows the calibration line used to convert the E_{diff} values from Figure 5b and c (main text) to BDD-Q pH values.

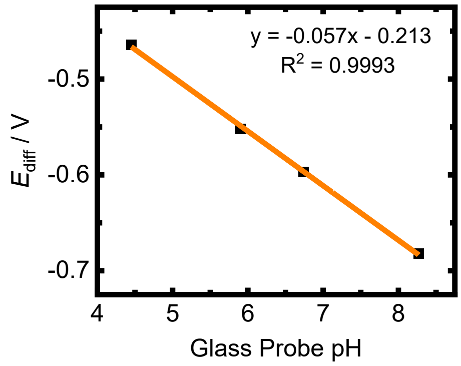

Figure S5. E_{diff} plotted against solution pH for Carmody buffer solutions of pH 4.45, 5.90, 6.74 and 8.27 with 0.1 M KCl

SI-9: Carbon Dioxide and Argon Compositions for CO_2 Experiment

100 sccm mass flow controllers were used to control the gas bubbled into a 20 mM HCO_3^- solution. The total gas flowed through the cell was kept constant (% CO_2 + % Ar = 100 sccm). The partial pressure of CO_2 (pCO_2) values are calculated using $pCO_2 = (760\ \text{mmHg} \times \%CO_2) \div 100$ where 760 mmHg is the total atmospheric pressure and $\%CO_2$ is the flow of CO_2 compared to the total gas flow through the cell. The experimental values used are given in **Table S5**.

Table S5. % CO_2, %Ar and resulting pCO_2 values in different gas mixtures.

% CO_2	% Ar	pCO_2 / mmHg
4	96	30.4
7	93	53.2
10	90	76.0
14	86	106.4
17	83	129.2
20	80	152.0

SI-10: CO_2 Calibration Curves

Figure S6 shows the calibration plots used to convert (a) E_{pH} and (b) E_{diff} to BDD-Q pH values. R^2 values > 0.99 are seen for both, with slopes of -56 and -61 mV / pH unit for E_{pH} and E_{diff} respectively. The expected Nernstian slope at 21°C is -58 mV / pH unit.

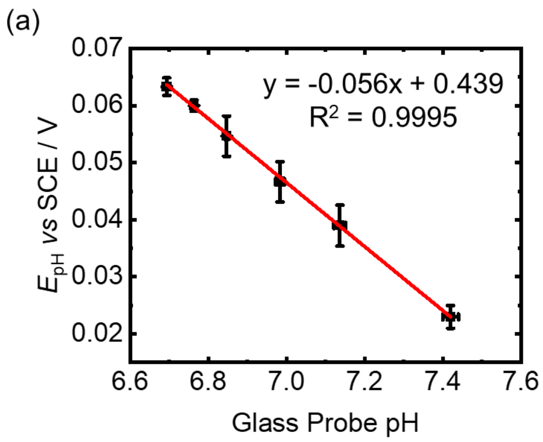

Figure S6. Calibration plot for BDD-Q calculated (a) using the E_{pH} peak and (b) with the use of the internal reference, and E_{diff}. n = 3 repeats on the same electrode.

SI-11: Potential *vs* log(pCO_2) Theory

Equation S1 shows the relationship between the potential ($E = E_{pH}$ or E_{diff} measured in volts, V) and pH when using a BDD-Q electrode at 25°C.[2] **Equation S2** shows a simplified version of the Henderson-Hasselbalch equation,[3–5] used to determine the relationship between pH and log(pCO_2). By substituting **Equation S2** into **Equation S1**, we can find the direct relationship between the potential and log(pCO_2), where a slope of 0.059 V (at 25°C) is observed (**Equation S3**). A, B, and C are constants.

$$E = -0.059\mathrm{pH} + \mathrm{A} \qquad [\mathrm{V}] \qquad \text{Equation S1}$$

$$\mathrm{pH} = -\log\left(\mathrm{pCO_2}\right) + \mathrm{B} \qquad \text{Equation S2}$$

$$E = +0.059\log\left(\mathrm{pCO_2}\right) + \mathrm{C} \qquad [\mathrm{V}] \qquad \text{Equation S3}$$

References

(1) Bard, A. J.; Faulkner, L. R.; White, H. S. *Electrochemical Methods: Fundamentals and Applications, 3rd ed.*; Wiley, 2022.

(2) Ayres, Z. J.; Borrill, A. J.; Newland, J. C.; Newton, M. E.; Macpherson, J. V. Controlled sp^2 Functionalization of Boron Doped Diamond as a Route for the Fabrication of Robust and Nernstian pH Electrodes. *Anal Chem* **2016**, *88* (1), 974–980. https://doi.org/10.1021/acs.analchem.5b03732.

(3) Severinghaus, J. W.; Bradley, F. Electrodes for Blood pO_2 and pCO_2 Determination. *J. Appl. Physiol.* **1958**, *13*, 515–520. https://doi.org/10.1152/jappl.1958.13.3.515.

(4) Delost, M. Blood Gas and Critical Care Analyte Analysis. In *Equipment for Respiratory Care*; 2014; pp 151–174.

(5) Severinghaus, J. W. CO_2 Electrodes. *Encyclopedia of Medical Devices and Instrumentation* **2006**. https://doi.org/10.1002/0471732877.emd326.
